# Influence of Morphology on the Healing Mechanism of PCL/Epoxy Blends

**DOI:** 10.3390/ma13081941

**Published:** 2020-04-20

**Authors:** Alberto Jiménez-Suárez, Gilberto Del Rosario, Xoan Xosé Sánchez-Romate, Silvia González Prolongo

**Affiliations:** 1 Area of Materials Science and Engineering, ESCET-University Rey Juan Carlos, c/Tulipán s/n, 28933 Móstoles, Madrid, Spain; alberto.jimenez.suarez@urjc.es (A.J.-S.); xoan.fernandez.sanchezromate@urjc.es (X.X.S.-R.); 2 Technological Center Support, University Rey Juan Carlos, c/Tulipán s/n, 28933 Móstoles, Madrid, Spain; gilberto.delrosario@urjc.es

**Keywords:** self-healing, epoxy blend, polycaprolactone

## Abstract

Polycaprolactone (PCL) is being researched as a self-healing agent blended with epoxy resins by several reasons: low melting point, differential expansive bleeding (DBE) of PCL, and reaction induced phase separation (RIPS) of PCL/epoxy blends. In this work, PCL/epoxy blends were prepared with different PCL ratios and two different epoxy networks, cured with aliphatic and aromatic amine hardeners. The curing kinetic affects to the blend morphology, varying its critical composition. The self-healing behavior is strongly affected by the blend morphology, reaching the maximum efficiency for co-continuous phases. Blends with dispersed PCL phase into epoxy matrix can also show high self-healing efficiency because of the low PCL domains that act as reservoir of self-healing agent. In this last case, it was confirmed that the most efficient self-healable blends are one whose area occupied by PCL phase is the largest. These blends remain the good thermal and mechanical behavior of epoxy matrix, in contrast to the worsened properties of blends with bicontinuous morphology. In this work, the self-healing mechanism of blends is studied in depth by scanning electron microscopy. Furthermore, the influence of the geometry of the initial surface damage is also evaluated, affecting to the measurement of self-healing efficiency.

## 1. Introduction

Currently, there is an increasing effort in avoiding corrective maintenance of a structure as a way to save costs. For this reason, the development of self-healing ability on thermosetting polymers is being widely researched for self-repairing damage and cracks [1,2]. There are several current approaches to achieve self-healing functionality on thermosetting resins [1]: (1) incorporation of microcapsules, which store the healing agent and usually the catalyzer; (2) modification of network formulation incorporating reversible covalent bonds, forming vitrimers; and (3) adding a second thermoplastic phase with relatively low melting point. All these strategies allow developing self-healable thermosets commonly thermal triggered. In this work, we focus the attention on thermoplastic/thermosetting (TP/TS) blends as they have proved good capabilities for self-healing purposes [3,4]. Around fifteen years ago, many works were published about the addition of TP into TS matrix to increase its toughness [5]. Poly(phenylene oxide), polysulfone, and polyetherimide, among others, were added to resins as reinforcing agents. The developed materials have currently numerous applications in different industrial fields. It is well known [5] that the initially soluble TP suffers a reaction induced phase separation (RIPS) during the crosslinking reaction of TS matrix providing biphasic morphologies. This phenomenon is explained by a loss of miscibility of the TP into growing TS network and therefore it depends on both a kinetic curing reaction and the thermodynamics of phase separation. The obtained TP/TS morphology is strongly influenced by TP fraction added. At contents lower than critical composition, the blend morphology is usually a dispersion of TP-rich domains in the TS matrix. However, at high TP percentages, a phase inversion occurs, showing TS-rich modules dispersed into a TP-rich matrix. Close to critical composition, the blend morphology can show co-continuous phases or doubled separated phases. For TS toughening, the desired morphology is the first one, TP domains dispersed into TS matrix [4,5]. The presence of low stiffness thermoplastic domains into brittle thermoset matrix induces the appearance of different toughening mechanisms, such as shear yielding, cavitation, crack deflection, crack bifurcation, and bridging, among others.

However, critical compositions are being more investigated [6], in the recent years, as self-healing strategy, due to the better distribution and higher area occupied by TP phase, which really acts healable agent.

Self-healing process is usually based on a mobile phase, triggered by external stimuli, which is able to transport melted TP mass to the crack site and local mending connection by chemical bonds or physical interactions. In the case of self-healable TS/TP blends, the healing process is based on the melting of the thermoplastic phase. This means that the main requirement of the TS/TP blend to show self-healing capability is the selection of a semicrystalline thermoplastic polymer, whose melting point (*T_m_*) is significantly lower than the glass transition temperature (*T_g_*) of the TS resin in order to promote enough flow to crack.

As it is mentioned above, several studies [1,2,3,4] indicate that the thermal healing of TS/TP blends is achieved at critical composition, with co-continuous morphology, when both components, TS and TP, form continuous phases. However, it is well known that these blends present weakened thermal and mechanical behavior. Toughened blends consist on TS matrix with dispersed TP phase, at TP concentration lower than the critical one.

Luo et al. [7] proposed, for the first time, a thermal healable epoxy blend with poly(caprolactone) (PCL), studying its mending adhesion ability at critical composition. Several years later, the same healable system was also studied by V. Michaud et al. [8] at different TP compositions. Other strategies, such as the use of shape memory TS matrix [9] or the addition of graphitic nanofillers [10,11] have been analyzed to enhance their self-healing efficiency while keeping the epoxy mechanical performance.

On the other hand, several years ago, looking for the development of toughened epoxy resins (ignoring their self-healing abilities), Chen and Chang [12] described in detail the dependence of PCL/epoxy blends on different amine hardeners, PCL composition and cure temperature. Grohens et al. [13] have recently published similar work about the influence of the curing agent used on the morphology and thermomechanical properties of these blends.

In the present study, we have investigated the phase separation, blend morphology, thermomechanical behavior, and self-healing efficiency of PCL/epoxy systems modifying the amine hardener and therefore the curing temperature and PCL composition. The selection of PCL as thermoplastic self-healing agent is justified by the PCL expansion within the cracks, which is not present in other semicrystalline thermoplastics. It is worthy to highlight that PCL/epoxy blends usually present high self-healing efficiency [3,14,15,16] due to their relative low triggering temperature, associated to the relative low melting point and differential expansive bleeding (DEB) of polycaprolactone during heating. On the other hand, the epoxy resins were cured with different amine hardeners, such as triethylenetetramine (TETA), with low curing temperature for sport and transport applications, and 4,4-diaminodiphenylsulfone (DDS), with high glass transition temperature, for aerospace applications [17]. Studied PCL/epoxy blends could be useful for developing self-healable coatings, with ability to repair surface cracks.

## 2. Experiment

### 2.1. Materials and Preparation of Samples

TP/TS blends based on epoxy/PCL were studied. Diglycidyl ether of bisphenol A (DGEBA), with an epoxide equivalent of 170.21 g/epoxy equivalent and a density of 1.16 g/cm^3^, was used as epoxy monomer. Triethylenetetramine (TETA) and 4,4’-diaminediphenylsufone (DDS), aliphatic and aromatic amines, with amine equivalent of 24.38 and 62.07 g/hydrogen amine equivalent, respectively, were added as curing agents. Polycaprolactone (PCL), whose average molecular weight was 14.000 g/mol, was added as self-healable and toughening thermoplastic agent. All these products were supplied by Sigma Aldrich (San Luis, MO, USA).

PCL was rightly dissolved into DGEBA at 80 °C. The homogenous mixture was degassed under vacuum conditions for 30 min to remove the entrapped air to avoid bubbles in the final cured blend. Then, the amine hardener was added at stoichiometric ratio. The curing treatment was different as a function of hardener. DGEBA/TETA was cured at 50 °C for 90 min and postcured at 150 °C for 30 min, while DGEBA/DDS required a curing treatment at 210 °C for 3 h. PCL was added in different percentages from 5 to 30 wt %, because P.T. Mather et al. [7] confirmed that the critical composition to obtain interconnected co-continuous double phase is close to 15% with DGEBA/DSS matrix.

### 2.2. Characterization

The morphology of PCL/epoxy blends was observed by Environmental Scanning Electron Microscopy (ESEM, Philips XL30, Amsterdam, Netherhands) and Field Emission Gun-SEM of high resolution (FEG-SEM, FEI, Nova NanoSEM 230, Hillsboro, OR, USA).The fracture surfaces were carried out at cryogenic conditions, using liquid nitrogen and they were coated with a thin sputtered Au(Pd) layer for a proper ESEM observation. The images were analyzed with specific images analysis software, named Scanning Probe Image Processor (SPIP)and ImageProPlus (IPP 5.1).

The characteristic transitions of polymeric components, Tm of polycaprolactone, and Tg of epoxy resins, were measured by differential scanning calorimetry (DSC, Mettler Toledo mod.822e, Columbus, OH, USA). The heating rate was 10 °C /min from 20 to 220 °C in nitrogen atmosphere.

Dynamic mechanical thermal analysis (DMTA) was carried out on a DMA Q800 V7.1 analyzer (TA Instruments, New Castle, DE, USA) with dual cantilever clamp. The applied frequency was 1 Hz while the test amplitude was 1% of the specimen thickness. The thermal scanning applied was from room temperature to 275 °C at 2 °C /min. The dimensions of the samples were 37.5 × 12 × 1.5 mm^3^.

The healing process was studied by promoting a controlled damage with a knife and analyzing it, before and after healing thermal treatment with an optical 3D perfilometer (Zeta Instruments, Phoenix, AZ, USA).

## 3. Results

### 3.1. Blend Morphology

**Figure 1:**
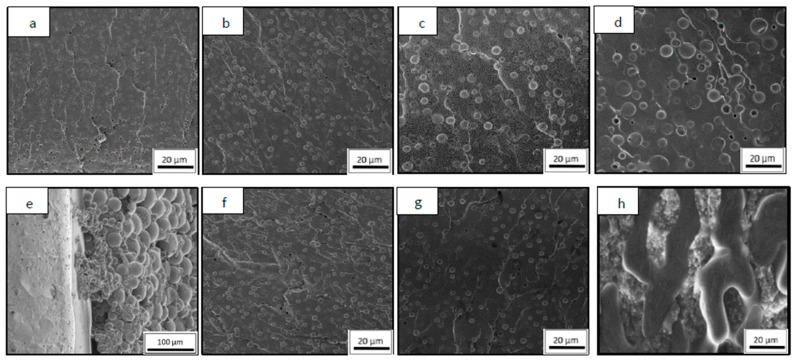
SEM images of PCL/epoxy blends: DGEBA/TETA resin modified with 10 (**a**), 15 (**b**), 20 (**c**), 25 (**d**), and 30 wt % PCL (**e**) and DGEBA/DDS resin with 10 (**f**), 15 (**g**), and 20 wt % PCL (**h**).

Figure 1 summarizes SEM images of PCL/epoxy blends, with DGEBA/DDS and DGEBA/TETA matrices. Only several studied blend compositions are shown as examples. In both studied cases, RIPS occurs, so two different phases can be seen: one corresponding to the rich-PCL phase and the other one to the epoxy matrix. There are numerous PCL/TS systems that prove totally miscible blends, forming inter-penetrating network (IPN), without phase separation [18,19]. This is explained because of the pendant hydroxyl groups, resulted from the oxirane ring opening reaction with the amine curing agent, which play an important role because they form hydrogen-bonds with PCL, enhancing its solubility into the epoxy network. However, in this work, PCL/epoxy blends cured with TETA or DDS present RIPS due to the high polarity of amine groups, which enhances the epoxy/amine reaction. The curing reaction is accelerated by the intramolecular bonds with the hydroxyl groups formed during curing process, limiting the intermolecular hydrogen bonds with PCL.

**Figure 2:**
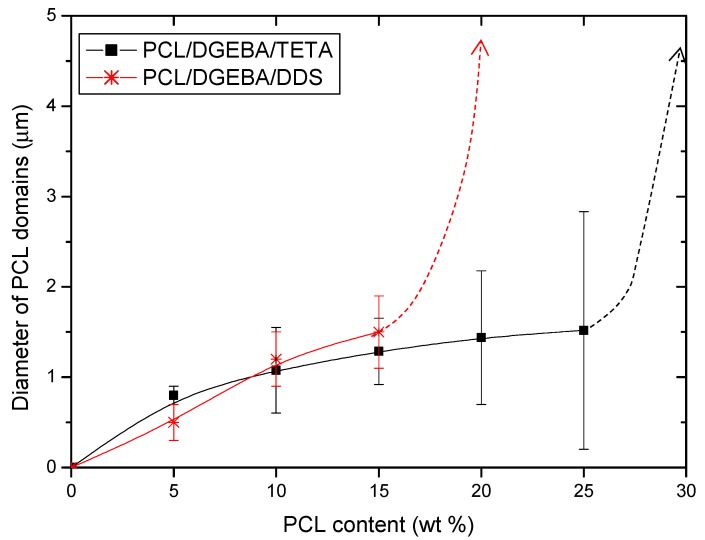
Average diameter of separated thermoplastic phase at PCL content lower than critical composition.

The morphology of PCL/epoxy blends with low thermoplastic contents is similar in both studied systems cured with different amine hardener. As it was expected, they present an epoxy matrix with dispersed spherical PCL phase, whose average diameter is shown in Figure 2.

However, when increasing PCL content, significant differences between PCL/DGEBA/TETA and PCL/DGEBA/DDS blends could be seen. More specifically, the composition at which the bicontinuous phase appeared, named critical composition, was quite different between both systems. This was close to 30 wt % for PCL/DGEBA/DETA blends while this was nearly half, 20 wt %, for PCL/DGEBA/DDS. It is worthy to indicate that the error bars of the measurements of PCL diameter (Figure 2) increased with the PCL content due to the higher heterogeneity of the separated thermoplastic phase at PCL percentages close to critical composition.

In general, the phase separation of blends occurred during the curing reaction because of the decrease of entropic contribution to free energy of mixing due to the increased molecular weight of the epoxy resin during its cure. The difference between both studied amine hardeners is that the sulfone groups of DDS are much stronger electron donors. This means that they preferentially form intramolecular hydrogen bonds with the epoxy hydroxyl groups, avoiding the intermolecular hydrogen bonds between PCL and epoxy, reducing the miscibility of PCL into epoxy resin. For this reason, the critical composition was much lower for the PCL/DGEBA/DDS system. This affirmation will be corroborated below by DSC analysis.

Bicontinuous TP/epoxy blend morphology (Figure 1e,h) was constituted by two main regions: (1) a continuous epoxy-rich phase with dispersed TP domains and (2) spherical particles of PCL-rich phase region.

**Figure 3:**
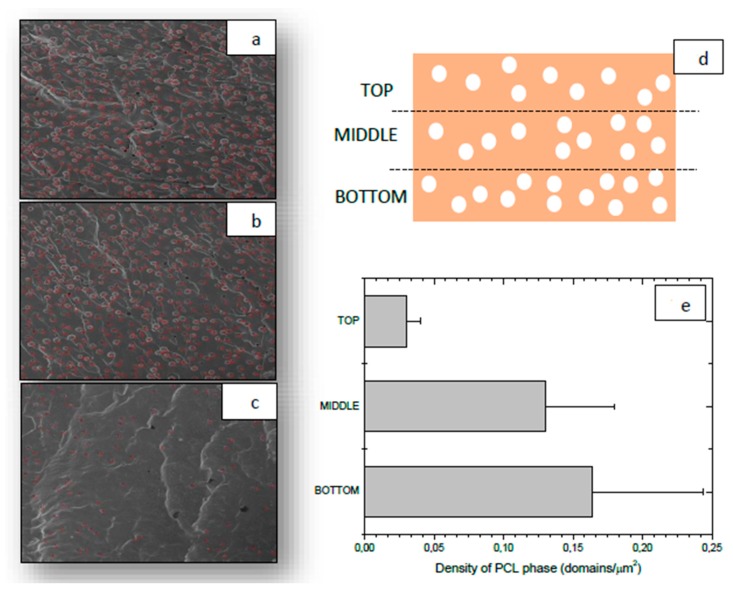
Analysis of PCL distribution for the PCL/DGEBA/TETA blend with 25 wt % PCL: SEM images at different location on the sample: top (**a**), middle (**b**), and bottom (**c**), scheme of studied areas (**d**) and density of separated PCL domains as a function of its location in the sample (**e**).

Nevertheless, both regions in PCL/DGEBA/DDS blends were uniformly mixed, in combined regions, homogenously distributed by the whole sample (Figure 1h), while in PCL/DGEBA/TETA blends, there was a clear phase segregation, being PCL-rich particles located at the bottom (Figure 1e). This sedimentation phenomenon was only observed for PCL/DGEBA/TETA blend at PCL contents nearby PCL critical composition (Figure 3). The sedimentation can be explained by the Stokes law:

(1) $$v_{s}=\frac{2}{9}\frac{r^{2}g\left(\rho_{p}-\rho_{r}\right)}{\eta }$$

where $v_{s}$ is the sedimentation speed, *r* is the particles equivalent ratio, $\rho_{p}$ and $\rho_{r}$ are the densities of the particles and resin, respectively, *g* is the gravity force, and η is the viscosity of non-cured resin.

When phase separation occurs at the cloud point, if there is a long time up to the gel point, the separated phases can sediment depending primarily upon the differences of densities between both phases, the size of separated phase and viscosity of mixture. The differences between densities are not noteworthy, being the density of thermoplastic polymer 1.146 g/cm^3^ while the density of epoxy phase varied during the curing reaction, from the density of mixture between non-cured DGEBA (1.16 g/cm^3^) and TETA (0.982 g/cm^3^) to the density of 1.124 g/cm^3^ for cured resin. Therefore, the main difference is the viscosity of mixture. The curing rate of DGEBA/TETA was much lower and therefore the viscosity of mixture will be lower for longer time [13], enhancing the PCL sedimentation.

Y. Grohens et al. [13] confirming that the phase separation on PCL/DGEBA/DDS blends happens at a relative higher conversion, higher than 50% [13], which prevents their sedimentation, since the gel point on epoxy/amine systems usually occurred close to 55% of the conversion. This means that the viscosity of PCL/DGEBA/TETA systems kept low for a longer curing time, up to the gel point.

This justifies the homogeneity of the PCL/DGEBA/DDS morphology in contrast to the sedimentation of PCL/DGEBA/TETA. The sedimentation was strongly observed on PCL/DGEBA/TETA blends at high PCL contents, close to critical content, where the concentration and size of PCL domains were higher (Equation (1)).

### 3.2. Thermal and Mechanical Behaviour

**Figure 4:**
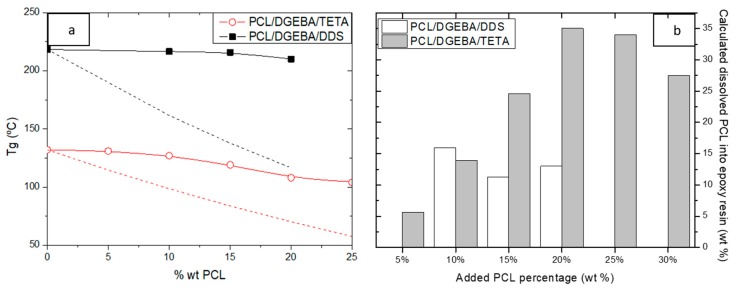
*T_g_* of epoxy resin as a function of PCL content added to the blend (**a**). Dot lines are the theoretical *T_g_* with total PCL miscibility. Estimated PCL amount dissolved into epoxy resin by the mix´s law (**b**).

DSC was used to determine the thermal behavior of studied blends. The first DSC scan firstly shows an endothermic peak, in the range of 57–59 °C, corresponding with the melting peak of PCL and therefore their enthalpy proportionally increased with PCL content added. Then, at higher temperatures, the glass transition of epoxy matrix appeared, whose values for different PCL/epoxy blends are collected in Figure 4.

*T_g_* of epoxy resin decreased with PCL content due to the partial PCL solubility into the thermosetting network. According to the Fox equation, the dissolved PCL remained approximately constant in PCL/DEGA/DDS blends (Figure 4b), being a low percentage, close to 10–15% regarding to the total PCL content added. However, the dissolved PCL amount in the DGEBA/TETA resin proportionally increased with the PCL percentage added to the blend, up to 35%. Then, the dissolved PCL again decreased near critical composition due to the inversion phases. The high PCL solubility into DGEBA/TETA was again explained by the higher chemical interaction, through hydrogen bonding, between hydroxyl groups of epoxy resin and polycaprolactone, which was minimized when DDS was used as a hardener.

**Figure 5:**
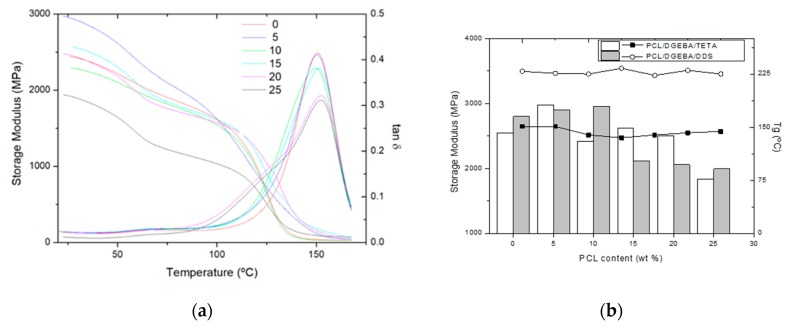
Dynamic mechanical thermal analysis (DMTA) curves of PCL/DGEBA/TETA blends (**a**) and DMTA results of PCL/epoxy blends (**b**): storage modulus in glassy state at 30 °C (bars in left axis) and α-relaxation temperature (dots in right axis).

Figure 5 collects the DMTA results. The DMTA test gave several curves, such as the storage modulus and loss tangent as a function of temperature, shown in Figure 5a for the PCL/DGEBA/TETA system with different PCL contents. The storage modulus is related to the stiffness of the sample, decreasing with the temperature up to a wide fall associated to α-relaxation of the major component, the epoxy resin. The tan delta curve represents the variation of the ratio between the storage and loss modulus, whose main peak indicates the α-relation transition. The peak related to the relaxation of epoxy resin widened with PCL contents due to the PCL solution. The glassy storage modulus at room temperature and the α-relaxation temperature of the epoxy resin, measured as the maximum of tan delta curve, are shown in Figure 5b for both studied blends. As it was expected, the relaxation temperature slightly decreased with PCL content, according to the tendency of the glass transition temperature measured by DSC. This decrease was associated to the partial miscibility of the thermoplastic into epoxy network, which was enhanced for the PCL/DGEBA/TETA system. In fact, the relaxation temperature of epoxy resin in the PCL/DGEBA/DDS system remained approximately constant, confirming the low solubility of polycaprolactone (10–15%).

The storage modulus seemed to remain almost constant for both blends, when they were constituted by an epoxy matrix with the dispersed TP phase. When the PCL content was close to the critical composition for both PCL/epoxy blends, the stiffness of the samples dropped off, due to the elastic contribution of the co-continuous PCL phase. In both systems, this fall occurred at PCL contents lightly lower to the critical composition, 15 wt % for PCL/DGEBA/DDS and 25 wt % for PCL/DGEBA/TETA.

**Figure 6:**
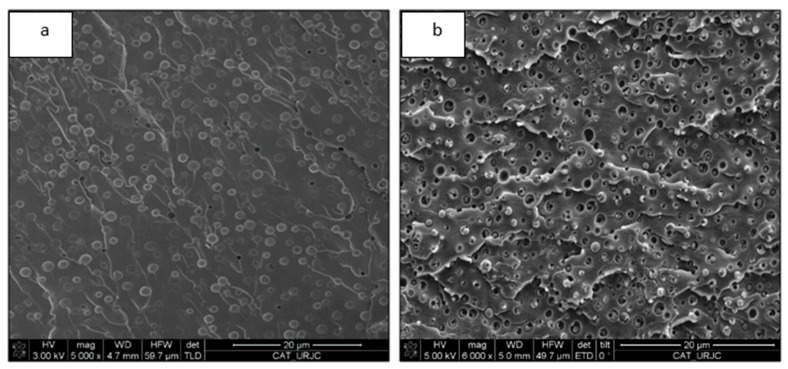
FEG-SEM micrographs of epoxy blends with 15% PCL: PCL/TETA/DGEBA (**a**) and PCL/DDS/DGEBA (**b**).

The addition of the thermoplastic or rubber phase into the epoxy thermosetting matrix usually produces a toughening of the brittle resin [5,8,20,21] due to the dissipation impact energy induced by the second low modulus polymer separated phase. The morphological study of the fractured surfaces on thermoplastic/thermosetting blends provides useful information about their mechanical behavior [20,21]. Figure 6 shows the fracture surfaces of epoxy blends doped with 15% PCL. In both cases, the surface shows a mostly smooth brittle fracture mechanism. The blend cured with DDS shows rougher fracture surfaces with several breaking planes, indicating higher toughness. The toughening mechanism of a thermoplastic toughened epoxy network was based mainly on the shear bands built when the stress field ahead the crack collide with thermoplastic particles. For this reason, the toughness improvement depended on the density of thermoplastic domains. In addition, other toughness mechanisms can also be operatives, such as crack deflection, bifurcation, bridging, or microcracking. The secondary toughening mechanism was different for both studied systems. The PCL/DGEBA/TETA sample presented whole PCL domains or several holes, observing crack defection (Figure 6a). However, the PCL/DGEBA/DDS blend shows all the thermoplastic particles broken with localized cavitation at the interface (Figure 6b). All these fracture mechanisms confirm the toughening of epoxy matrix by the addition of polycaprolactone.

### 3.3. Self-Healing Properties

**Figure 7:**
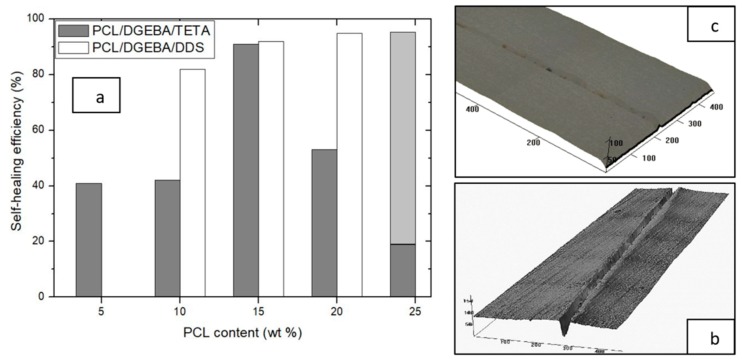
Self-healing efficiency of PCL/epoxy blends (**a**). Images of optical perfilometry for non-repaired (**b**) and self-healed cracks (**c**) for DGEBA/DDS blend reinforced with 15% PCL.

The self-healing mechanism on PCL/epoxy blends consists on the thermoplastic melting and its flow to fill the crack, enhanced by the low melting point of PCL, 69 °C (measured by DSC), and its differential expansive blending (DEB). The most efficient self-healable system is usually an interconnected and interpenetrated thermoplastic and thermosetting material [2]. However, these blends, with co-continuous phase morphology, usually present diminished thermomechanical properties (Figure 5) together with a higher morphological heterogeneity (Figure 1). For this reason, in this study, the self-healing efficiency was analyzed for blends with different PCL contents. Figure 7 shows the self-healing efficiency of the studied blends calculated as the ratio of depths of the self-healed and the neat crack. Self-healing process was triggered at 90 °C for 2 min. In spite of the lower melting temperature of polycaprolactone, 68 °C, the minimum temperature to induce an efficient self-healing was fixed at 90 °C, at which the thermoplastic can flow due to a lower viscosity.

As it was expected, PCL/DGEBA/DDS blend with co-continuous phases (20 wt % PCL) was the most efficient as a self-healable material. On the other hand, the anomalous efficiency of PCL/DGEBA/TETA blend with 25 wt % PCL was explained by the thermoplastic phase sedimentation (Figure 3), causing a high variability on the measurements of healing efficiency, from around 20–90%. It depended on the measured face of the specimen, top and bottom side, which had low or high PCL domains concentration, respectively.

Both studied systems also show high self-healing efficiency at lower PCL content. In particular, the self-healing efficiency of both epoxy resins with 15 wt % PCL was unexpectedly high, despite its morphology, constituted by separated PCL domains into epoxy matrix. This behavior is very interesting since the TS/TP blends keep the excellent behavior of the epoxy matrix with enhanced toughness [8] associated to thermoplastic domains and, in addition, it presents an efficient self-healing ability. In order to explain this last behavior, a digital image analysis of FEG-SEM was carried out. The results are collected in Table 1, where the concentration and size of PCL domains are shown as a function of the PCL content added.

It is confirmed that at lower PCL content than critical composition, the concentration of separated PCL domains initially decreased while their size grew with the increment of PCL percentage on the blend. The PCL/epoxy blend with the highest area occupied by the self-healing agent, PCL, was the sample reinforced with 15 wt % for both studied systems, which explains its highest self-healing efficiency. Again, the anomalous low area occupied by PCL domains in the PCL/DGEBA/DDM blend doped with 25 wt % PCL was due to the sedimentation, measuring two different PCL concentration on the upper and bottom side of the specimen.

**Figure 8:**
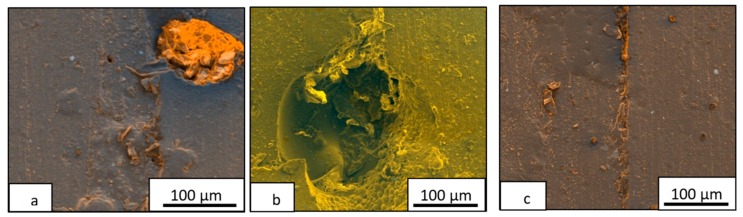
FEG-SEM images to analyze the self-healing mechanism of PCL/DGEBA/TETA blends with 10% (**a**), 25% (**b**), and 15% PCL (**c**).

The repaired cracks were observed by FEG-SEM, which are shown in Figure 8, in order to analyze the self-healing mechanism. At low PCL content, the dispersed PCL phase acted as a reservoir of the self-healing agent to fill the crack (Figure 8a). However, it could be observed that the self-healed crack had a different nature than the matrix. The crack was filled with PCL while the matrix was the epoxy resin. It is worthy to note that this self-healing thermoplastic crystallized in granules during the cooling after the thermal healing applied. For this reason, this cooling would be controlled to avoid it. This phenomenon will be studied in future works. When a separated PCL phase grew, at PCL percentages close to critical one, it was possible to observe holes (Figure 8b) provide by the PCL flow.

These evidences mean that when the morphology is constituted by epoxy resin with dispersed PCL domains, there is an optimum size of PCL domains to act as an efficient healing agent. This content was 15 wt % for both systems, confirming their highest self-healing efficiency (Figure 7 and Table 1). Finally, Figure 7c shows an area with a total repaired crack with scarcely any differences between the matrix and crack at critical PCL content.

These observations allow affirming that the study of the self-healing process though filling of cracks, which is common in many published works, was not enough. The measurement of depth only quantifies the amount of the self-healing agent into the crack but a deeper study of the quality of this healing is required, evaluating the filling material and the surroundings.

**Figure 9:**
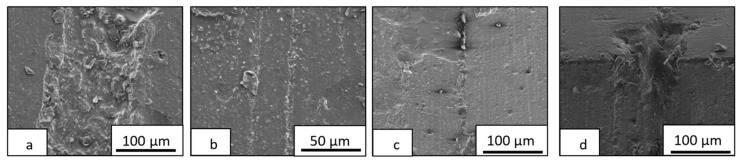
FEG-SEM images of self-healed samples with different initial width of crack close to 160 µm (**a**), 38 µm (**b**), and 10 µm (**c**); and the end of the crack (**d**).

Finally, the influence of the geometry of the initial crack was also studied (Figure 9). The induced cracks were always superficial, with an average depth close to 70 µm. Not great differences were observed on the self-healing efficiency as a function of the crack depth at least in the studied range. However, the width of the crack, from ten to hundred microns, significantly affected the self-healing efficiency, as it is shown in Figure 9. This implies that a standard test should be proposed in order to compare self-healing efficiencies between systems published by different researchers.

In studied PCL/epoxy blends, a suitable self-healed material was obtained when the width of the crack was lower than a hundred microns (Figure 9b,c). On the other hand, it was confirmed that the self-healing efficiency was lower at the end of the crack (Figure 9d), where more healing time or temperature was required.

## 4. Conclusions

PCL/epoxy blends were studied as self-healable materials. Their thermal, mechanical, and self-healing properties strongly depended on the blend morphology, which, in turn, was influenced by the curing kinetic of the epoxy resin. This means that the curing hardener used significantly affected the behavior of these materials. DGEBA cured with an aromatic amine (DDS) presents a low PCL miscibility and, therefore, the *T_g_* of epoxy matrix remained constant and the critical composition at which the phase inversion occurred was low (20 wt % PCL). DGEBA cured with aliphatic amine (TETA), at low temperature, enhanced the PCL miscibility due to the intermolecular hydrogen bonding between PCL and hydroxyl groups of epoxy network. This induced a decrease of *T_g_* of epoxy resin and an increment of PCL critical composition (30 wt % PCL). The phase separation on PCL/DGEBA/DDS blends occurred close to the gel point, avoiding the PCL sedimentation, which was clearly observed in PCL/DGEBA/TETA blends at PCL percentages near to critical composition.

Without PCL sedimentation, the self-healing efficiency was high when the blends morphology was co-continuous. However, the stiffness of these materials was low. On the other hand, it is worthy to note that PCL/epoxy blended with a specific low PCL content, 15 wt %, also presented high self-healing efficiency for both studied resins in spite of their morphology consisting of separated PCL-rich domains into an epoxy-rich matrix. This was explained due to the highest ratio of area occupied by the PCL phase, which acts as repository of self-healing agent.

In addition, it was also confirmed that the geometry of the damage modifies the measurement of self-healing efficiency. This means that standardized tests should be proposed in the future.

## Figures and Tables

**Table 1:** Image analysis results of blends morphology.

Hardener	PCL * Content (wt %)	Average Diameter of PCL Domains (µm)	Average Area of PCL Domains (µm^2^)	Number of PCL Domains per Sample Area (domains/µm^2^)	Percentage of Sample Area Occupied by PCL (%)
TETA	10	1.08 ± 0.47	0.912	0.162	14.8
	15	1.29 ± 0.38	1.299	0.158	20.5
	20	1.44 ± 0.74	1.624	0.113	18.3
	25 **	1.52 ± 1.31	1.814	0.04–0.17	7.2–30.8
DDS	5	0.64 ± 0.25	0.321	0.142	4.5
	10	1.12 ± 0.52	0.985	0.166	16.3
	15	1.38 ± 0.38	1.495	0.293	43.8

* PCL domains cannot be measured at critical composition due to the phase inversion, at 30% PCL for PCL/DGEBA/TETA and 20% PCL for PCL/DGEBA/DDS blend. ** Due to the PCL sedimentation (Figure 3), the PCL concentration is measured on the bottom and the top side of specimen.
